# Thermal energy storage and thermal conductivity properties of fatty acid/fatty acid-grafted-CNTs and fatty acid/CNTs as novel composite phase change materials

**DOI:** 10.1038/s41598-020-71891-1

**Published:** 2020-09-21

**Authors:** Amir Al-Ahmed, Ahmet Sarı, Mohammad Abu Jafar Mazumder, Billel Salhi, Gökhan Hekimoğlu, Fahad A. Al-Sulaiman, Inamuddin

**Affiliations:** 1 Center of Research Excellence in Renewable Energy (CoRERE), King Fahd University of Petroleum & Minerals, Dhahran, 31261 Saudi Arabia; 2 Department of Metallurgical and Material Engineering, Karadeniz Technical University, 61080 Trabzon, Turkey; 3 Chemistry Department, King Fahd University of Petroleum & Minerals, Dhahran, 31261 Saudi Arabia; 4 Advanced Functional Materials Laboratory, Department of Applied Chemistry, Faculty of Engineering and Technology, Aligarh Muslim University, Aligarh, 202 002 India

**Keywords:** Chemistry, Energy science and technology

## Abstract

In recent year, fatty acids (FAs) are heavily studied for heat storage applications and they have shown promising advantages over other organic phase change materials (PCMs). Among the FAs; capric, palmitic and stearic acids are the most studied PCMs. Several researchers have investigated these FAs and tried to improve their thermal properties, mainly by adding different high conducting fillers, such as graphite, metal foams, CNTs, graphene etc. In most cases, these fillers improved the thermal conductivity and heat transfer property but reduce the heat storage capacity considerably. These composites also lose the mixing uniformity during the charging and discharging process. To overcome these issues, selected FAs were grafted on the functionalized CNT surfaces and used as conductive fillers to prepare FA based composite PCMs. This process significantly contributed to prevent the drastic reduction of the overall heat storage capacity and also showed better dispersion in both solid and liquid state. Thermal cycling test showed the variations in the thermal energy storage values of all composite PCMs, however, within the tolerable grade and they had appreciable phase change stability and good chemical stability even after 2,000 cycles.

## Introduction

Utilization of heat energy using phase change materials (PCMs) is an economical and environment friendly approach^1^. Among the different PCMs, there is a long list of organic compounds which have been studied for latent heat thermal energy storage (LHTES)^2,3^. These PCMs do not suffer from super cooling, they are chemically stable, and possess non-toxic/non-corrosive characteristics^2^. Among the organic PCMs, fatty acids (FAs) are extensively studied in recent years, due to their several superior properties over the other organic PCMs, such as, melting congruency, good chemical stability, nontoxicity, suitable melting temperature range and remarkable latent heat capacity (LHC) for solar passive heating applications^4,5^. Like all other organic PCMs, FAs also suffer from low thermal conductivity (TC) and leakage issue, if not properly housed. Easy and cheaper solution is the composite formation with suitable filler. A suitable filler based composite can enhance the heat conduction ability, and at the same time can provide stable form i.e. housing of the FAs. In a form-stable structure, we can avoid additional cost of encapsulation and can have wide dimensional flexibility. Different conductive filler have been tried^6,7^, however multiwall carbon nanotubes (MWCNTs here termed as CNTs only) are the favourite for TC enhancement of organic PCMs due to their excellent thermal and mechanical properties and stability^8–11^. Recently, Yadav et al.^12^ studied capric acid (CA) mixed with three different (0.01, 0.02 and 0.025) volume % of CNTs, and evaluated their thermo-physical parameters using T-history method. Among these three composites, 0.02 vol% of CNTs containing samples were found to be the best composition in terms of storage enthalpy, thermal cycling and stability. Liu et al.^13^ prepared a shape stabilized phase change material (SSPCM) by impregnating CA and CNTs within the fly ash. The composite sample showed much lower latent heat capacity as compare to that of the pure CA but it was comparable with the other reported CA based composite PCMs^13^. However, the heat transfer efficiency of the composite samples was found to be much higher than that of pure CA. In another study, Yang et al.^14^ prepared composite PCMs with different CNT contents (2, 6 and 10 wt%). Like all other previous studies, the latent heat capacity decreases with the increasing amount of CNTs but the TC increases, so do the heat release rate.

The addition of pure carbon-based nano fillers to organic PCMs was responsible for the non-homogenous dispersion, which ultimately influence all thermal features of the final composite. Another issue was observed that the LHC of the PCM decreases with the increasing amount of carbon fillers, however the TC increases. Li et al.^15^ impregnated stearic acid (SA) within acid treated CNTs in different wt%. Composite samples showed a great reduction in melting/freezing temperature of SA due to the interface confinement effect. However, its melting LHC was decreased with the increasing the amount of CNTs. Wang et al.^16,17^ modified CNTs by four different methods, namely, acid oxidation, mechano-chemical reaction, ball milling and grafting following acid oxidation. The modified CNTs were then mixed with palmitic acid (PA), and ultra-sonicated to get homogeneous distribution. It was observed that the latent heat of the composite PCM decreases with the increasing amount of CNTs, but again the TC increases. TC was found to be 51.6% higher with the addition of only 1 wt% of CNTs. In another work, the same research group^18^ also grafted oleylamine and octanol on the acid treated CNTs and used as a filler to prepare paraffin wax/CNTs composite PCMs. They showed that the latent heat capacity of the composite PCMs was also decreased with the increasing amount of CNTs, but TC increases. Moreover, the octanol grafted CNTs based composites showed higher TC values compare to that of oleylamine grafted CNTs based composites. Ji et al.^19^ used acid treated CNTs and pyrogallo absorbed CNTs to prepare composite PCMs with PA, and observed similar trend, the thermal conductivity increases with the increasing amount of the filers. Xiao et al.^20^ dispersed CNTs, oxidized CNTs and grafted CNTs into PA at a mass ratio of 1:100. The LHC of PA/G-CNTs were found to be exceeded that of pure PA and showed a higher TC. Shen et al.^21^ modify multi-walled CNTs and dispersed them into erythritol as PCM. They observed that this treatment improved the dispersion of CNTs in the melted PCM and TC value of the PCM was significantly boosted by adding 1.0 wt% CNTs. Li et al.^22^ grafted CNTs with octanol, tetradecyl alchohol and stearyl alcohol and then added to paraffin. The results demonstrated that the dispersion ability of grafted CNTs was better than CNTs and the TC of the grafted CNTs/paraffin composite PCMs was relatively higher than that of CNTs/paraffin.

As seen from the above summarized literatures that only in few studies modified/or grafted CNTs were used to improve the thermal properties of the FAs by minimizing the agglomeration problem of CNTs. However, the effect of the grafting amount of CNTs on the LHTES, cycling thermal reliability and thermal degradation stability as well as the TC of FAs were not investigated. With this sense, in the present work, three major FAs commonly used as PCM, namely capric, palmitic and stearic acids (CA, PA and SA) were selected. As different from the literature, these FAs were grafted on the functionalized CNTs (CNTs-*g*-FA) in three different grafting ratios and then the grafted CNTs were added to the FAs in ratio of 5 wt% to achieve thermally enhanced composite PCMs. For comparison, a composite of FAs with non-grafted CNTs additive in the same weight amounts was also prepared. The prepared FA/CNTs-*g*-FA and FA/CNTs composite PCMs were characterized and evaluated for morphology, distortional homogeneity and thermal properties. The obtained results were compared systematically to exhibit the effects of the grafted and non-grafted CNTs additives on the dispersion stability, phase change temperatures/LHC properties, TC values, cycling thermal/chemical stability and high thermal degradation temperatures of the FAs. The findings indicated that the FAs doped with CNTs-*g*-FA additives had better dispersion stability (both solid and melted state), higher LHC and TC values compared to FA/CNTs composites.

## Experimental

### Materials

Capric acid (CA; purity degree: ≥ 98%; melting point: 27–32 °C), palmitic acid (PA; purity degree: ≥ 98%; melting point: 62–65 °C) and stearic acid (SA; purity degree: ≥ 98%; melting point: 67–72 °C) were obtained from Merck company (Germany), and used without further purifications. Thionly chloride (SOCl_2_) and nitric acid (HNO_3_) were purchased from BDH Chemicals Ltd. The CNTs were supplied from Cheap Tubes Inc. (Grafton, USA).

### Grafting of CNTs on FA (CNTs-*g*-FA)

Reported literature based procedure^22,23^, was used to acid functionalize CNTs. Required amount of CNTs (5 g) were added to 350 mL of HNO_3_ (60% v/v) solution in a round bottom flask and then stirred at room temperature for 3 h. The reaction mixture was refluxed at 120 °C for 12 h, and then cool to room temperature. It was filtrated via vacuum filtration, and the filtrate was washed with de-ionized water until a neutral pH was obtained. These functionalized CNTs (CNTs-COOH) were dried in vacuum oven at 60 °C until constant weight. In the second step of synthesis, CNTs-COOH (4 g) were mixed slowly with 60 mL SOCl_2_ in a 250 mL round bottom flask at room temperature. After that, the reaction mixture was refluxed at 80 °C for 24 h. Finally, extra SOCl_2_ was distilled off and the product was washed with de-ionized water to achieve acyl chloride-functionalized CNTs (CNTs-COCl).

**Figure 1:**
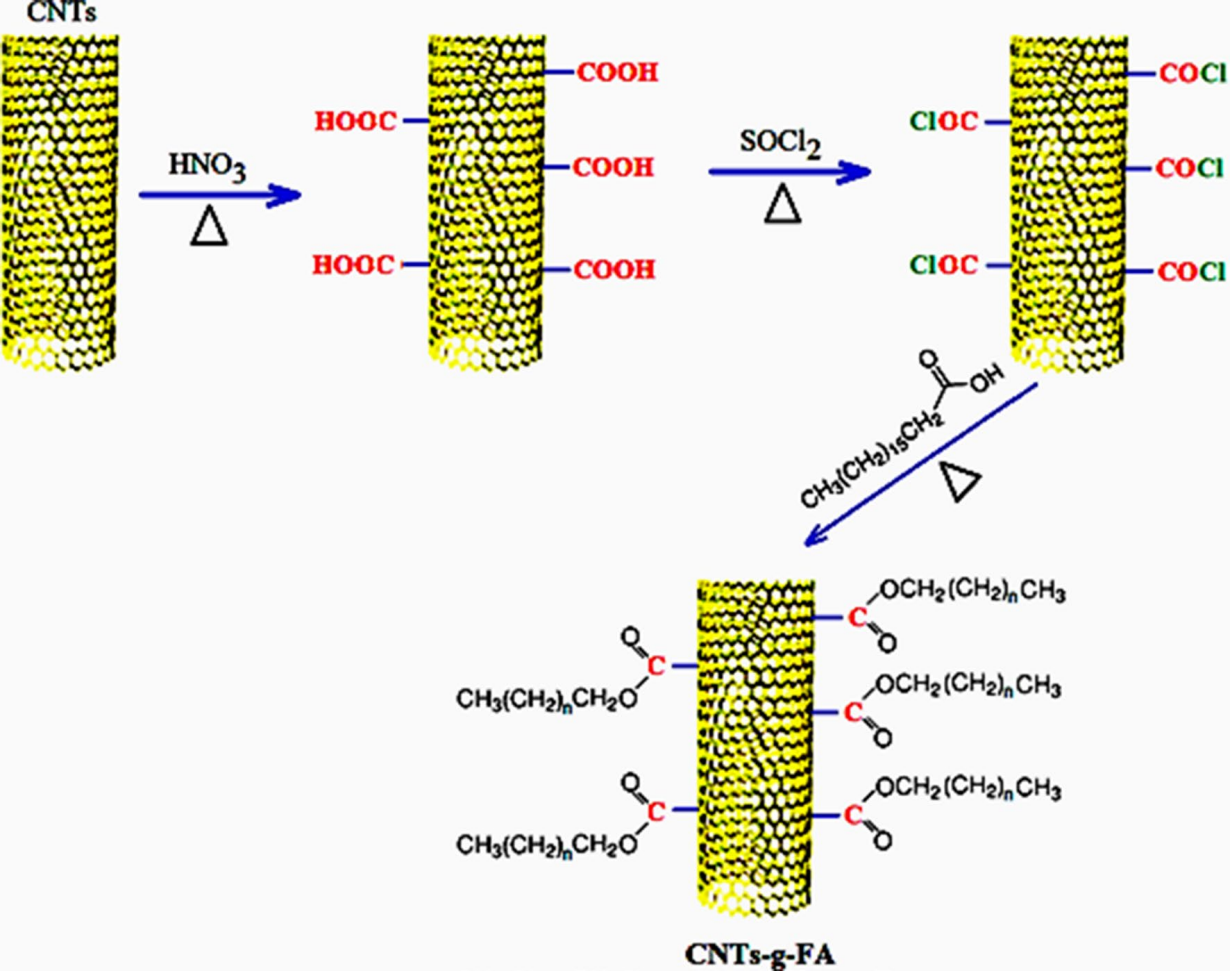
Synthesis/preparation schema for CNTs-*g*-FA.

In the final step, a specific amount of selected FA was melted and combined with the CNT-COCl at a ratio of 2:2 (w/w) into a round bottom flask. The mixture was refluxed at 75 °C for 48 h with stirring at 100 rpm. To remove the unreacted FA, the product (CNTs-*g*-FA) was washed carefully, and finally dried in vacuum oven for 24 h. The above-mentioned procedure was repeated to synthesis CNTs-*g*-FA samples for the other weight ratios of CNTs:FA as 3:1, and 1:3 (w/w). The reaction scheme used for the synthesis of CNTs-COOH, CNTs-COCl and CNTs-*g*-FA products is indicated in Fig. 1.

### Preparation of FA/CNTs-g-FA and FA/CNTs composites

To prepare FA/CNTs-*g*-FA composite samples*,* required amount of FA was melted and slowly mixed with CNTs-*g*-FA (grafting ratio of 2:2) in weight fraction of 95/5 (%) using a controlled shaker at 200 rpm for 30 min. After thorough mixing, the composite sample was cool to room temperature and stored in a glass bottle for further use. The same method was used to fabricate the other composites using CNTs-*g*-FA with grafting ratio of 3:1 and 1:3 (w/w). For comparison, composite samples with pure CNTs (FA/CNTs) were prepared using the same procedure, as described above. According to grafting ratio, 3:1, 2:2 and 1:3(w/w), the synthesized grafted products were called as CNTs-*g*-FA(3–1), CNTs-*g*-FA(2–2) and CNTs-*g*-FA(1–3), whereas the fabricated composite PCMs were named as FA/CNTs-*g*-FA1, FA/CNTs-*g*-FA2 and FA/CNTs-*g*-FA3, respectively. Moreover, the names of the composite samples doped with non-grafted CNTs were shortened as FA/CNTs. The selected FAs were doped with the same amount (5 wt%) of CNTs-*g*-FA and CNTs additives, separately.

### Dispersion stability experiment

To observe the dispersion behavior of the grafted and non-grafted CNTs in FA, the melting/blending method was conducted. As a representative example, the dispersion of CNTs-*g*-PA and CNTs in PA was carried out separately. The PA sample at specific amount was melted in two glass tubes at 70 °C. The CNTs-*g*-PA and CNTs powders were added in weight fractions of 5 wt% to the liquid PA samples. The obtained suspensions were centrifuged at 100 rpm for 20 min to achieve uniform dispersion. Afterwards, PA/CNTs-*g*-PA and PA/CNTs samples were kept at 70 °C for 30 min, and studied the dispersion stability.

### Characterization methods

The chemical and crystalline structures of the synthesized CNTs-*g*-FA, FA/CNTs-*g*-FA and FA/CNTs samples were studied by Fourier-transform infrared (FTIR; Perkin Elmer, 16F PC) and X-ray powder diffraction (XRD) instrument (PANalytical X'-Pert3 model). The FTIR spectra were obtained in wavelength range of 4,000–400 cm^−1^. The crystalline structures of the samples were identified using Cu (Kα = 1.5406 Å) irradiation at a step size of 0.0131°. The morphological structures of CNTs-*g*-FA, FA/CNTs-*g*-FA, and FA/CNTs samples were investigated using a scanning electron microscope (SEM; LEO 440 model).

The TES properties of the synthesized CNTs-*g*-FA, FA/CNTs-*g*-FA and FA/CNTs samples were measured by differential scanning calorimetry (DSC; Hitachi-7020) at a heating/cooling rate of 3 °C min^−1^. The analyses were repeated for three times to minimize the data error. The average deviation in phase change temperature and latent heat capacity (LHC) measurements was determined as ± 0.11 °C and ± 1.15%, respectively. Thermal degradation temperatures of the prepared CNTs-*g*-FA, FA/CNTs-*g*-FA and FA/CNTs samples were determined by thermogravimetric analysis (TGA) technique (Perkin–Elmer). The analysis was performed at heating ramp of 10 °C min^−1^ in temperature range of 25–580 °C. The degradation temperature data were determined with average discrepancy of ± 1.08 °C. To study the influence of the thermal cycling process on the chemical stability and phase change properties of the prepared FA/CNTs-*g*-FA and FA/CNTs composites, an accelerated heating–cooling process was adapted to the samples using a thermal cycler (Model: BIOER TC-25/H). This treatment was continued until the cycling number reached to 2000. After cycling procedure, FTIR, and DSC analyses were repeated on the studied samples. Thermal conductivity (TC) of CNTs-*g*-FA, FA/CNTs-*g*-FA and FA/CNTs samples as well as pure CA, PA and SA were measured at 20 ± 1 °C using a thermal property analyzer (Decagon KD2 pro Devices, Inc., USA). The measurements were repeated three times and the maximum discrepancy was determined as ± 0.02 W m^−1^ K.

## Results and discussion

### Microstructural and dispersion stability study

**Figure 2:**
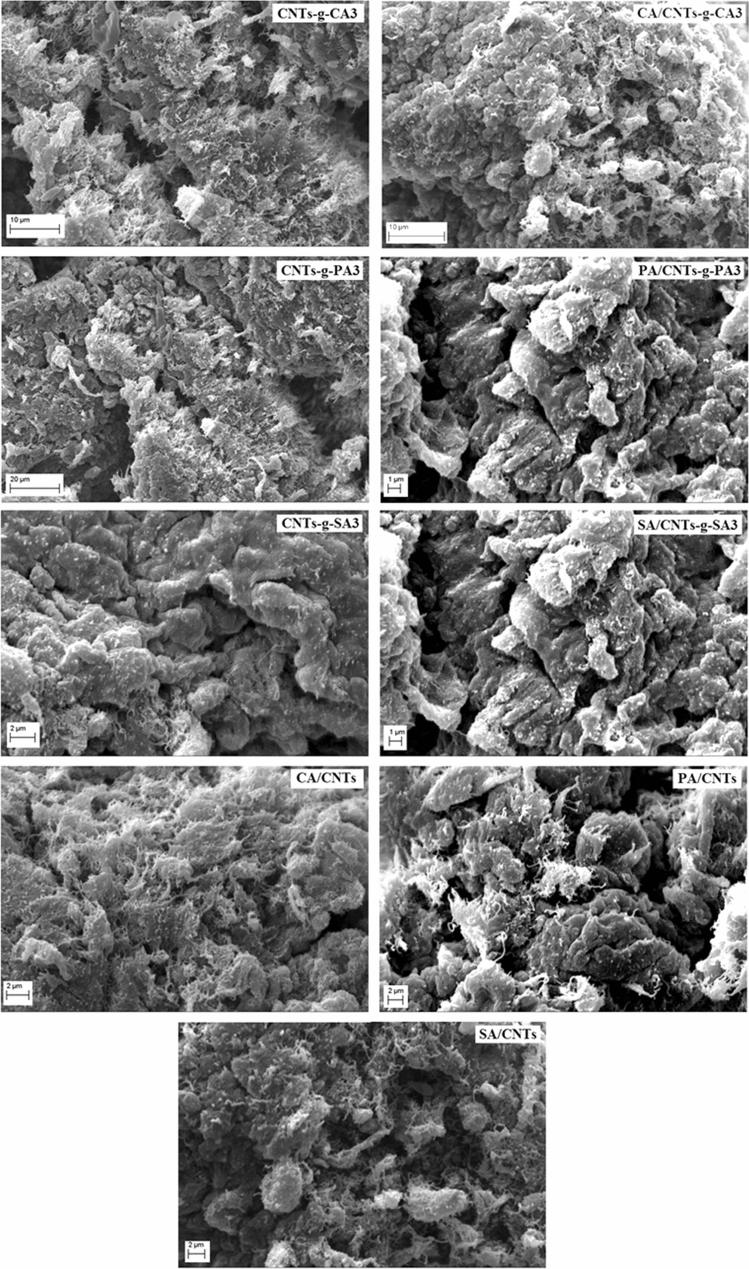
SEM images of CNTs-*g*-FA, FA/CNTs-*g*-FA and FA/CNTs composites.

Surface morphology of the CNTs-*g*-FA, FA/CNTs-*g*-FA and FA/CNTs composites were studied by SEM analysis. The obtained SEM microstructures are shown in Fig. 2. It is clear from the images (Fig. 2) that the CNTs-*g*-FA are uniformly distributed within the FA/CNTs-*g*-FA composites structure without agglomeration. Moreover, the nanotube phases are more visible on the surface of FA/CNTs composites. It can be assumed that this uniform distribution of the CNTs (both chemically grafted and pure) within the composites creates effective heat conduction path.

**Figure 3:**
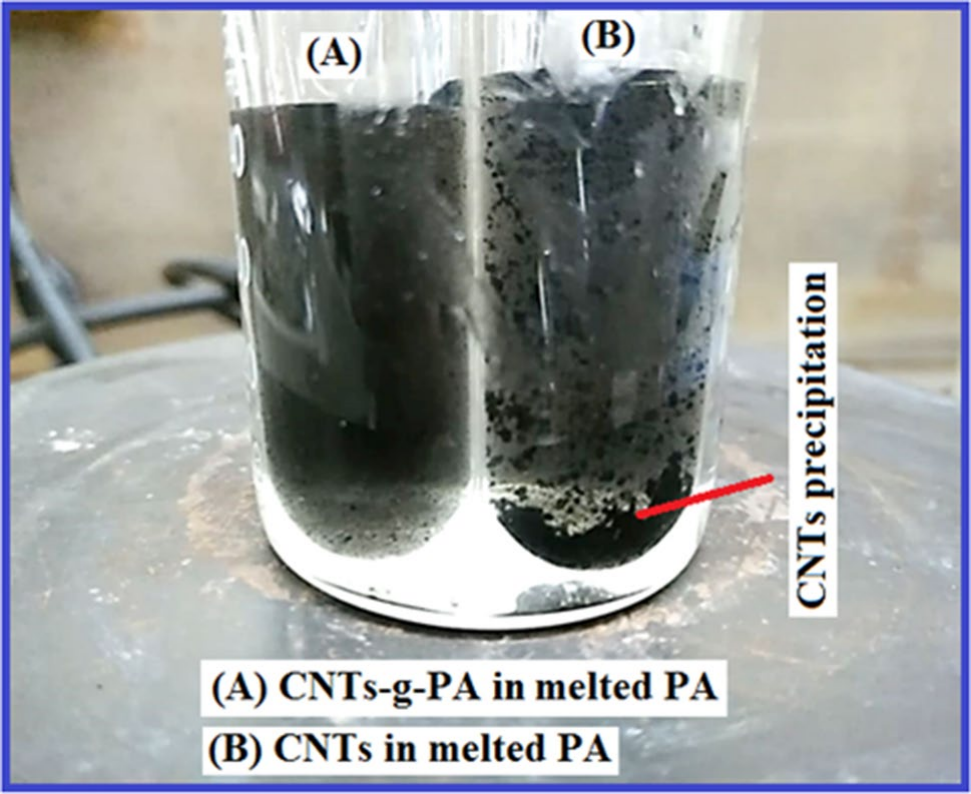
Photograph image of PA/CNTs-*g*-PA (5 wt%) and PA/CNT (5 wt%) suspensions after 30 min-standby at 70 °C.

Figure 3 exhibits the photographic image of the dispersion study of the grafted and non-grafted CNTs in melted PA as a representative example of other composites. This photograph was taken for PA/CNTs-*g*-PA (5 wt%) and PA/CNTs (5 wt%) composites samples at 70 °C after 30 min-standby period. As evidently perceived (Fig. 3), some CNTs precipitation was occurred in PA/CNTs sample while the PA/CNTs-*g*-SA (5 wt%) had a stable suspension. This result suggested that the grafted CNTs provide better dispersion stability, as compare to that of non-grafted CNTs. It can be directed to the comparatively stronger colloidal interaction between the CNTs-*g-*FA and FA. The similar observation was reported for acid modified CNTs/erythritol suspension^21^ and CNTs-*g*-polyalcohol/paraffin suspensions^22^.

### Chemical and crystalline characterization

**Figure 4:**
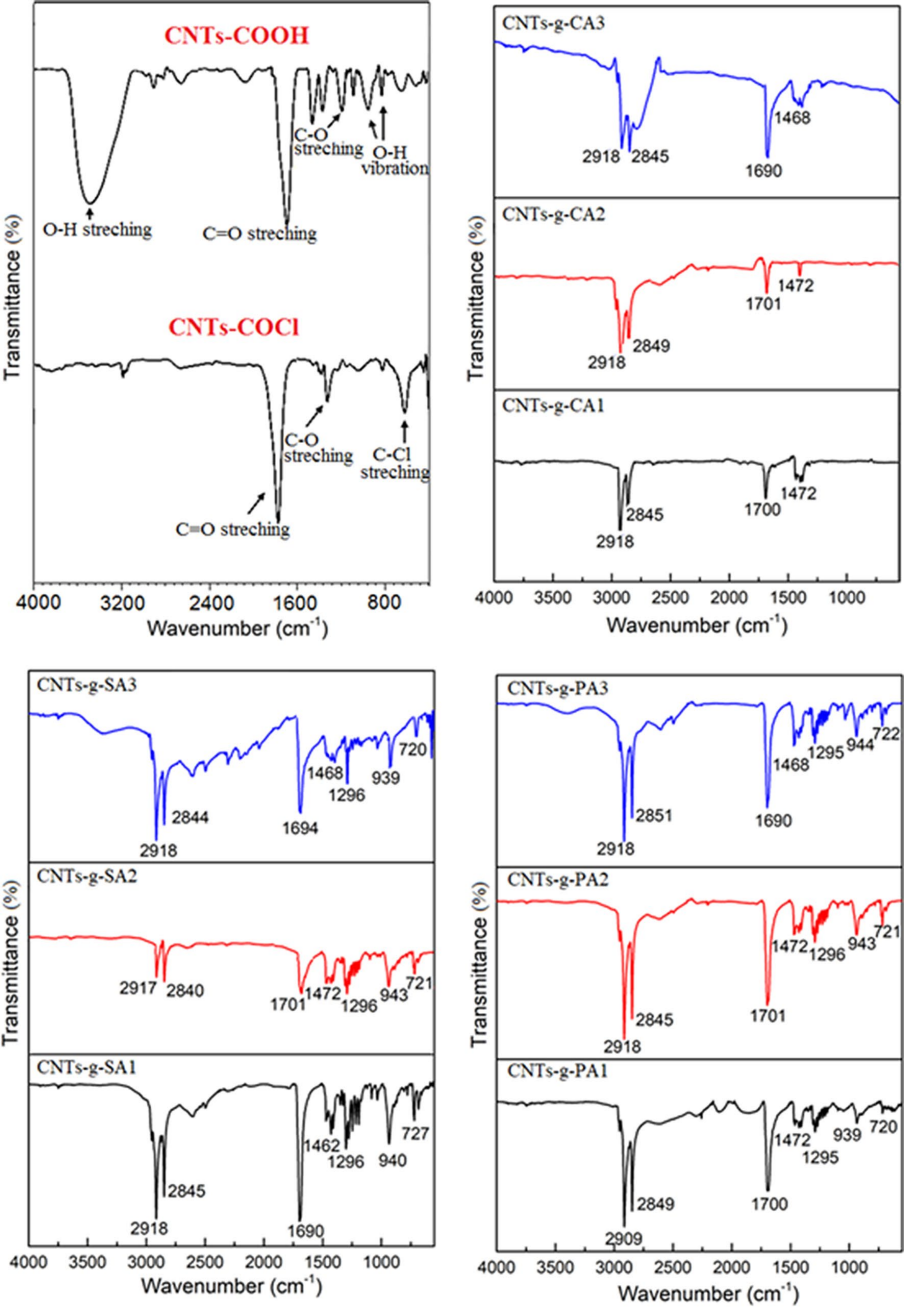
FTIR spectral results of CNTs-*g*-FA.

The FTIR results of the carboxylic acid functionalized CNTs (CNTs-COOH), acyl chloride functionalized CNTs (CNTs-COCl) and fatty acid functionalized CNTs (CNTs-*g*-FA) are shown in Fig. 4. In the spectrum of CNTs-COOH, the wide peak at 3250–3650 cm^−1^, signified the vibration band of –OH groups^24^. The peak at 1690 cm^−1^ is associated with the stretching band of C=O^25^ as the peaks at 958 cm^−1^ and 829 cm^−1^ are linked with the bending vibration band of –OH groups^26^. In the spectrum of CNTs-COCl, the peak at 1776 cm^−1^ is associated with the stretching band of C=O group. The peaks at 1328 cm^−1^ and 639 cm^−1^ are due the C–O and C–Cl groups of acyl chloride functionalized CNTs, respectively.

On the other hand, in the spectra of CNTs-*g*-FA compounds synthesized at three different grafting ratios, the peaks observed in the range of about 2840–2918 cm^−1^ are characterized as the stretching band of –CH_2_ and –CH_3_ groups. The C=O and C–O stretching bands were shifted to the range of 1295–1296 cm^−1^ and 1690–1700 cm^−1^ after grafting reaction. The intensity of –OH absorption observed at 3400–3650 cm^−1^ was considerably reduced due to grafting reaction, although it was seen in spectrum of CNTs-*g*-SA3 and CNTs-*g*-PA3 synthesized at highest grafting mole ratio (FA/CNTs mole ratio: 3:1). The similar observations were reported for grafted lauric acid with graphene oxide^26^. Moreover, the disappearance of the C–Cl absorption band in all spectra of CNTs-*g*-FA compounds also confirmed that the grafting reaction between FA and CNTs-COCl was successfully carried out. As a whole, all of these results shown in Fig. 4 confirm that the FAs were successfully grafted to the CNTs.

**Figure 5:**
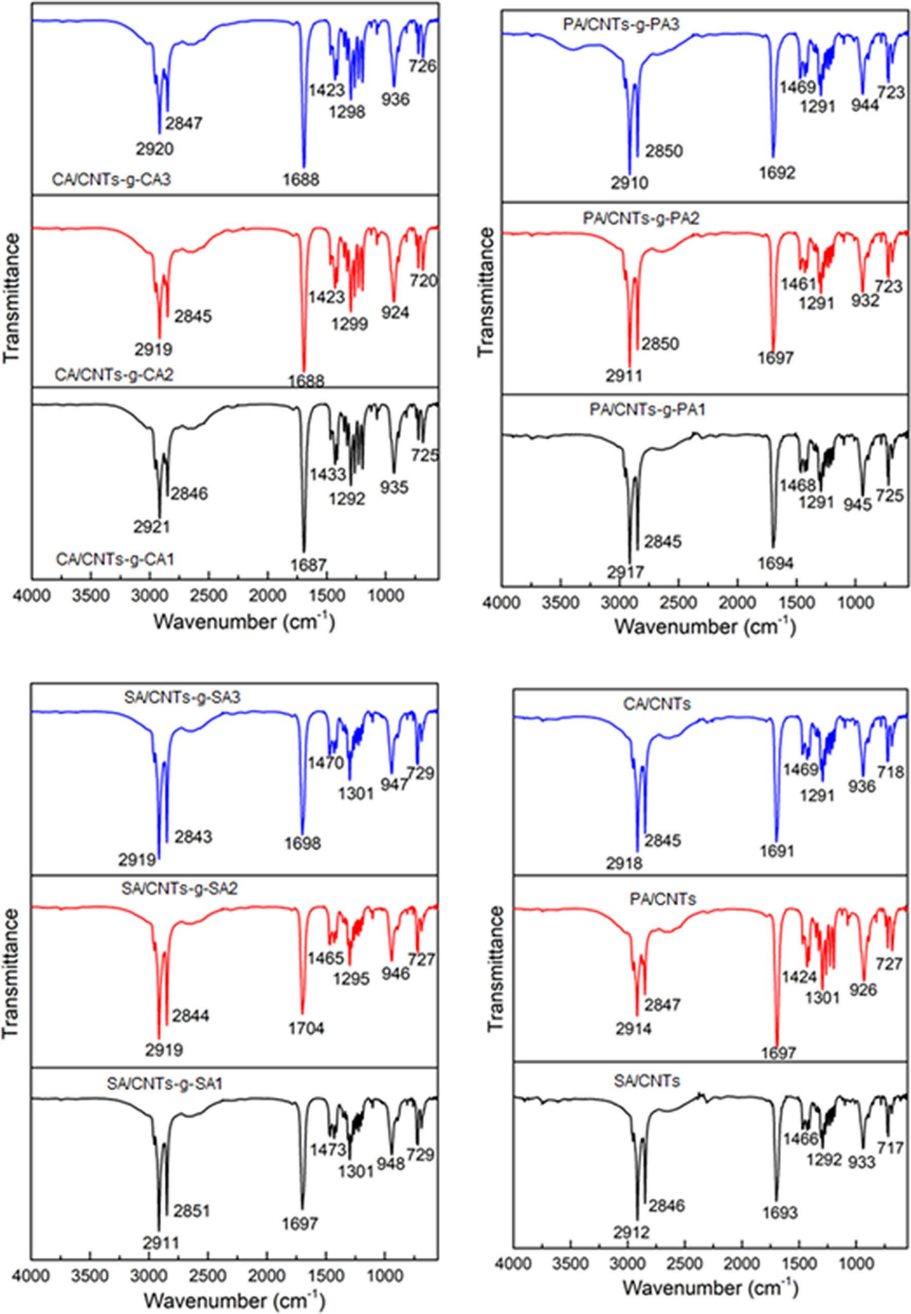
FTIR spectral results of FA/CNTs-*g*-FA and FA/CNTs composites.

Figure 5 shows the FTIR spectra of the prepared FA/CNTs-*g*-FA and FA/CNTs composites. The peaks between 2943–2920 cm^−1^ and 2945–2918 cm^−1^ are associated with the stretching band of methyl and methylene groups of nine different FA/CNTs-g-FA composites and three different FA/CNTs composites, respectively. The peaks around 1687–1704 cm^−1^ and 1691–1697 cm^−1^ are related with their C=O stretching vibration bands. Moreover, the stretching and bending vibrations belong to C–O and C–H band are detected in the spectra of the composites. When compared to the spectra of CNTs-*g*-FA, the FTIR spectra of the composites does not show any significant shift of the respective key peak position. This result proved that the presence the good chemical compatibility among FAs and CNTs-*g*-FA or CNTs.

**Figure 6:**
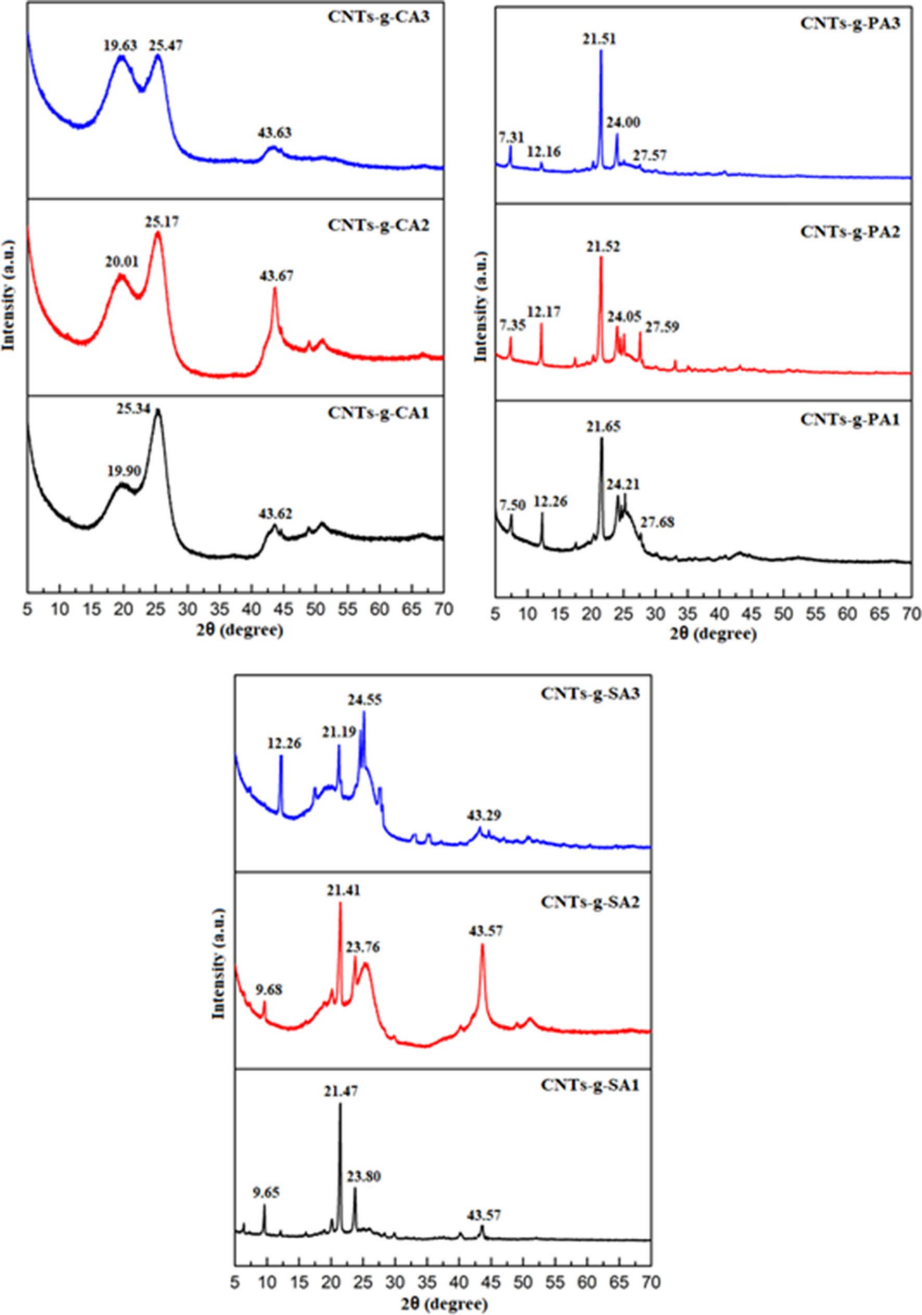
XRD results of CNTs-*g*-FA.

**Figure 7:**
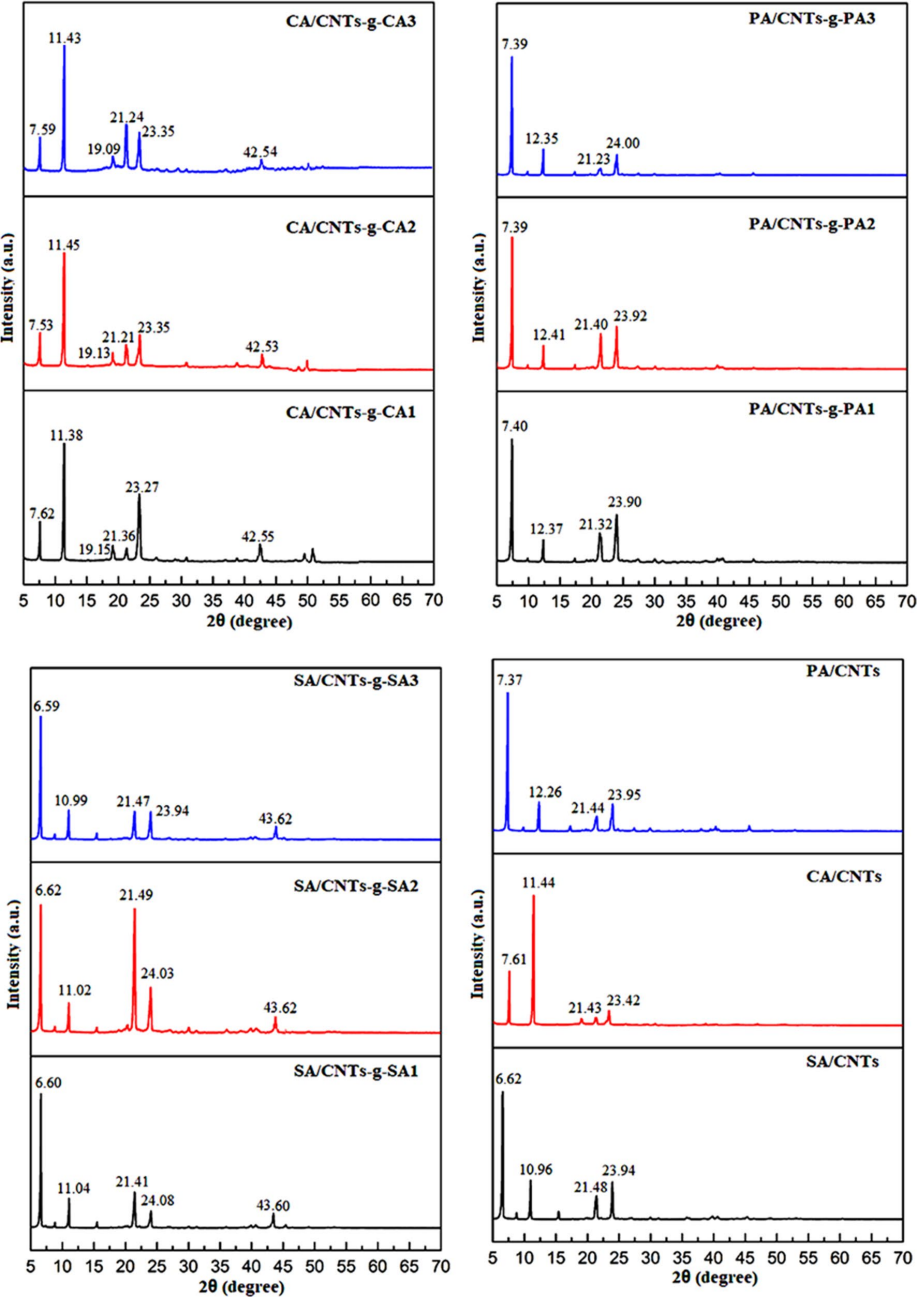
XRD results of FA/CNTs-*g*-FA and FA/CNTs composites.

On the other hand, the XRD analysis of the prepared FA/CNTs-*g*-FA and FA/CNTs composites were carried out to examine the influence of added CNTs-*g*-FA and CNTs on the crystal structures of SA, PA and CA. Figure 6 shows the XRD diffraction patterns of CNTs-*g*-CA, CNTs-*g*-PA and CNTs-*g*-SA samples. While, the XRD results of CA/CNTs-*g*-CA, PA/CNTs-*g*-PA, SA/CNTs-*g*-SA and CA/CNTs, PA/CNTs and SA/CNTs composites were presented in Fig. 7. The CA, PA and SA grafted CNTs has typical crystalline peaks in 2*θ* range of 19.63°–43.67°, 7.31°–27.68° and 9.68°–43.57°, respectively. The characteristic peaks were detected at 7.58°–23.38° for CA/CNTs-*g*-CA, 7.39°–24.0° for PA/CNTs-*g*-PA, 6.59°–24.08° for SA/CNTs-*g*-SA while they are observed at 7.61°–23.42° for CA/CNTs, 7.37°–23.95° for PA/CNTs and 6.62°–23.94° for SA/CNTs composites. Moreover, it is hard to notice the distinctive crystalline peaks of grafted or non-grafted CNTs because their amounts are quite low in the composites^27^.

### LHTES properties

**Figure 8:**
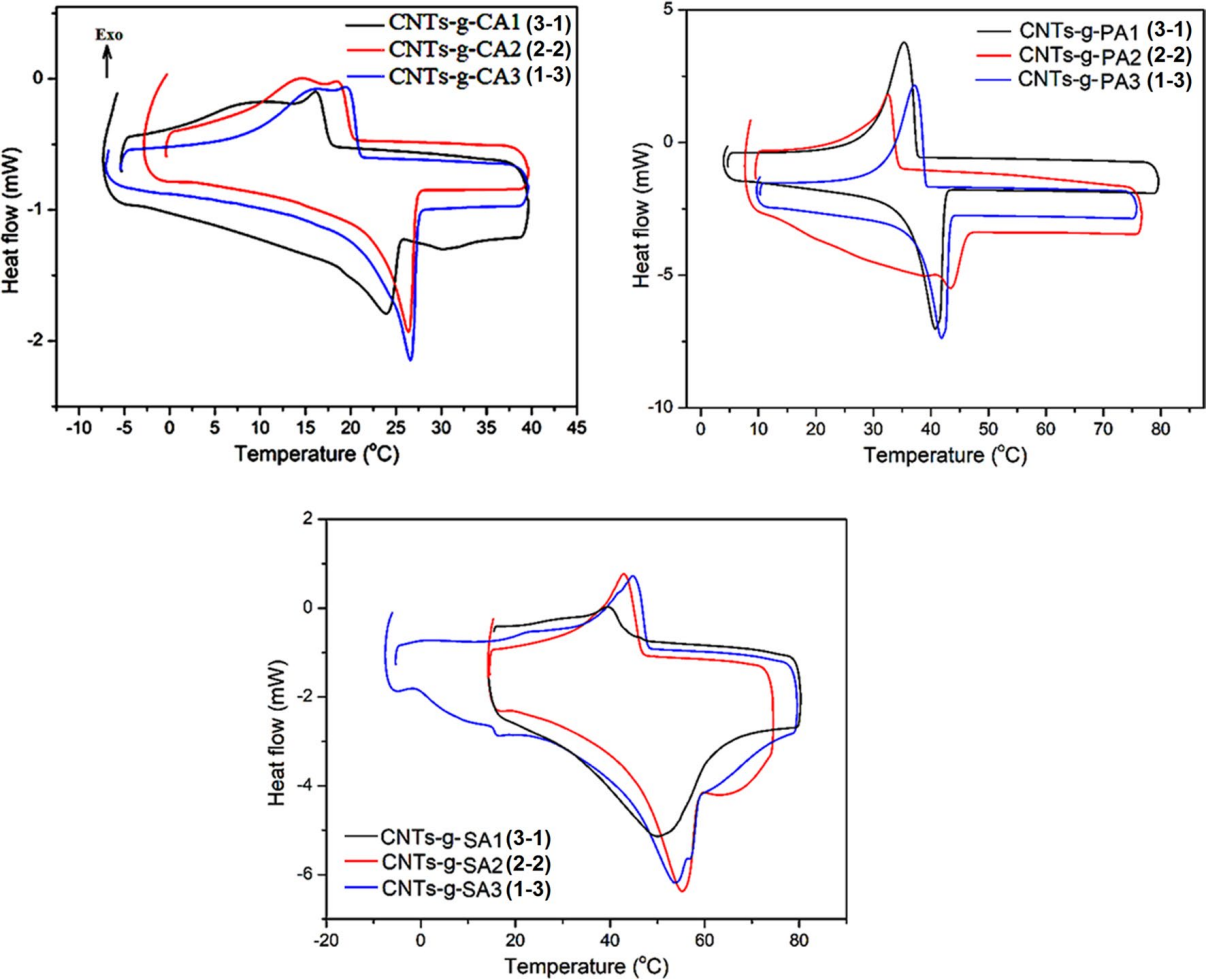
DSC thermograms of CNTs-*g*-FA.

**Figure 9:**
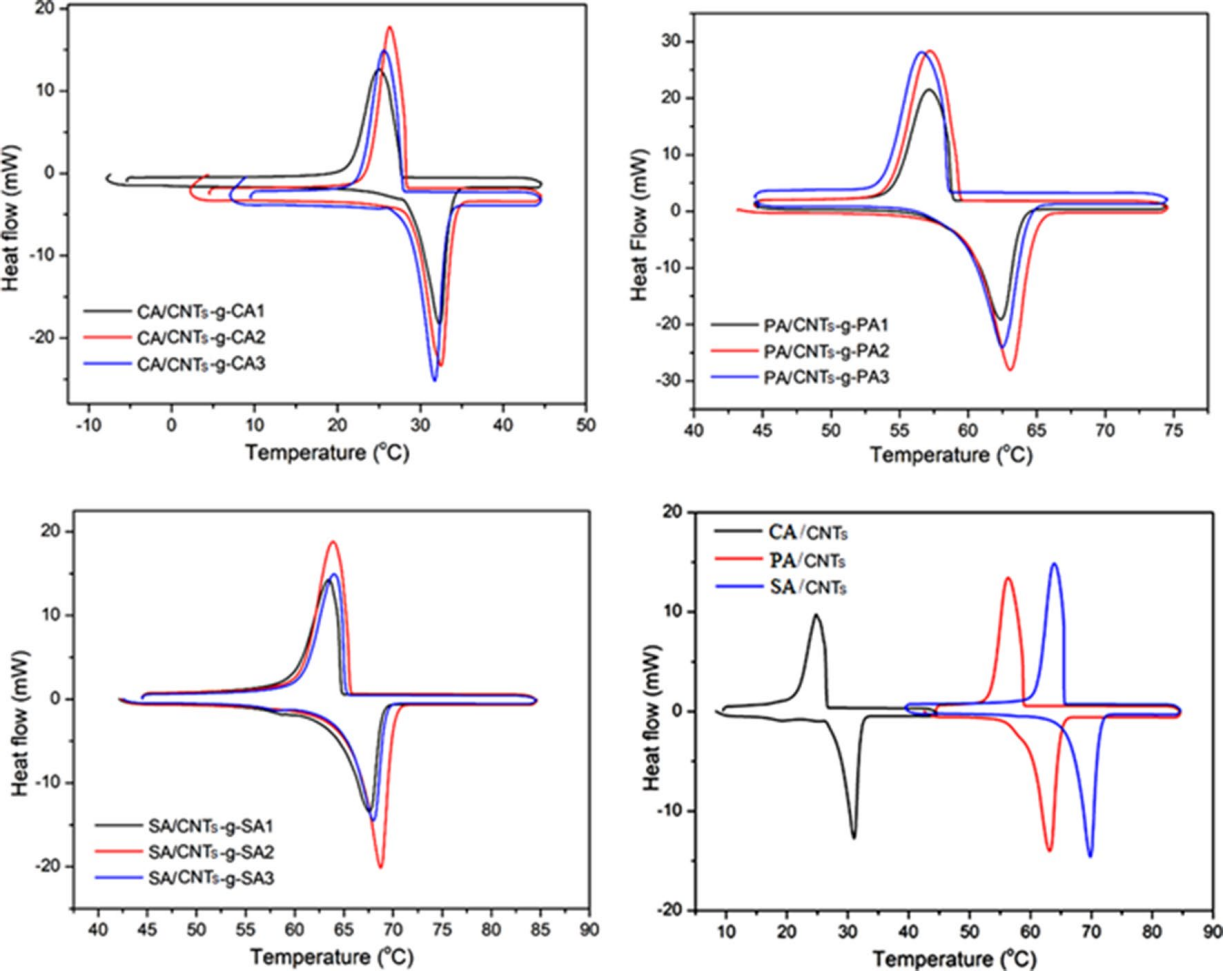
DSC thermograms of FA/CNTs-*g*-FA and FA/CNTs composites.

The integration of a PCM with a supporting material causes a significant reduction in the latent heat capacity (LHC) of the composite PCM. However, an adequately high LHC is one of the elementary selection criteria for composite PCMs and phase transition behaviors are crucial parameters to estimate the LHTES properties of composite PCMs. In this regard, the LHTES properties of CNTs-*g*-FA, FA/CNTs-*g*-FA and FA/CNTs composites were analyzed by DSC, and presented in Figs. 8 and 9, and data tabulated in Tables 1 and 2. From these data, it can be clearly observed that the melting and solidification temperatures of the FA were not changed regularly while the melting and freezing LHCs of the composite samples enhanced as expected. The melting temperatures of the CNTs-*g*-FA were changed in the range of 22.44 ± 0.12–23.35 ± 0.12 °C for CNTs-*g*-CA, 36.39 ± 0.11–39.41 ± 0.11 °C for CNTs-*g*-PA and 38.62 ± 0.12–40.43 ± 0.10 °C for CNTs-*g*-SA. The phase change temperatures of the composites are very different from those of pure CA (27–32 °C), PA (62–65 °C) and SA (67–72 °C) due to the change in the heat flow path and mechanism with these new compounds. Depending on the increase in the grafting ratio of FA, the latent heat of fusion of the CNTs-*g*-FA was increased from 24.62 ± 0.23 to 83.60 ± 0.85 J g^−1^ for CNTs-*g*-CA, 24.21 ± 0.26 to 87.93 ± 0.88 J g^−1^ for CNTs-*g*-PA and 27.25 ± 0.25 to 93.17 ± 0.97 J g^−1^ for CNTs-*g*-SA. In addition, the melting temperatures of the composites including 5 wt% grafted compounds were measured in the range of 28.85 ± 0.10–29.43 ± 0.13 °C for CA/CNTs-*g*-CA, 59.42 ± 0.10–60.0 ± 0.10 °C for PA/CNTs-*g*-PA and 63.82 ± 0.11–64.90 ± 0.10 °C for SA/CNTs-*g*-SA composite. The significant decrease in the melting temperatures of the composite PCMs in comparison with those of pure FAs could be due to the dispersion of CNTs grafted with long-chain FA molecules among the FA crystals. Moreover, the melting temperature was determined as 28.07 ± 0.10 °C for CNTs/CA, 59.70 ± 0.12 °C for CNTs/PA and 66.5 ± 0.11 °C for CNTs/SA. The phase changes temperatures of the composites including non-grafted CNTs are relatively closer to those of the pure FAs given above. This means that the physical attractions (e.g., capillary and surface tension forces) between the FA and grafted CNTs are more effective compared to the attractions between FA and FA-*g*-CNTs. Again, depending on the increase in the grafting ratio of FA, the melting LHCs of the composites were determined, which was in the range of 174–184 J g^−1^ for CA/CNTs-*g*-CA, 214–230 J g^−1^ for PA/CNTs-*g*-PA and 235–257 J g^−1^ for SA/CNTs-*g*-SA. Whereas the melting LHC of CNTs/CA, CNTs/PA and CNTs/SA was found to be about 171, 210 and 234 J g^−1^, respectively. As seen from these data, especially the LHC values of the composites including CNTs-*g*-FA (grafting ratio: 1:3) were 6.5–8.9% higher than that of the composites including CNTs due to the LHC contribution of the CNTs-g-FA additive. On the other hand, when compared the LHC of the FA/CNTs-*g*-FA composite PCMs with those of different kinds of organic PCMs doped with carbon based fillers^14–20,22,26,29,36^, it was observed that the FA/CNTs-g-FA composite PCMs prepared in this work had relatively higher LHC than most of them. This boosted in the LHC value of the FA/CNTs-FA composite PCMs was due to the fact that the CNTs-*g*-FA used as doping agents had LHC of in the range of 25–93 J g^−1^.

**Table 1:** The LHTES properties of synthesized CNTs-*g*-FA.

Material	Melting temperature (°C)	Solidification temperature (°C)	Melting enthalpy (J g^−1^)	Solidification enthalpy (J g^−1^)
CNTs-*g*-CA1	22.44 ± 0.12	18.03 ± 0.10	24.62 ± 0.23	− 20.57 ± 0.22
CNTs-*g*-CA2	23.35 ± 0.11	20.55 ± 0.12	58.34 ± 0.57	− 52.74 ± 0.53
CNTs-*g*-CA3	22.00 ± 0.10	21.10 ± 0.11	83.60 ± 0.85	− 75.69 ± 0.78
CNTs-*g*-PA1	36.39 ± 0.11	36.69 ± 0.13	24.21 ± 0.26	− 23.85 ± 0.21
CNTs-*g*-PA2	39.41 ± 0.11	33.99 ± 0.10	60.24 ± 0.62	− 54.66 ± 0.56
CNTs-*g*-PA3	37.28 ± 0.12	38.99 ± 0.10	87.93 ± 0.88	− 83.95 ± 0.81
CNTs-*g*-SA1	38.62 ± 0.12	43.93 ± 0.10	27.25 ± 0.25	− 26.22 ± 0.27
CNTs-*g*-SA2	40.43 ± 0.10	45.47 ± 0.11	63.14 ± 0.61	− 57.40 ± 0.58
CNTs-*g*-SA3	40.41 ± 0.10	48.26 ± 0.12	93.17 ± 0.91	− 92.13 ± 0.91

**Table 2:** The LHTES properties of FA/CNTs-*g*-FA and FA/CNTs composites.

Material	Melting temperature (°C)	Solidification temperature (°C)	Melting enthalpy (J g^−1^)	Solidification enthalpy (J g^−1^)
CA/CNTs-*g*-CA1	28.85 ± 0.10	28.07 ± 0.11	174.12 ± 1.73	− 173.65 ± 1.76
CA/CNTs-*g*-CA2	29.43 ± 0.13	28.57 ± 0.13	179.03 ± 1.81	− 178.76 ± 1.83
CA/CNTs-*g*-CA3	28.84 ± 0.12	27.95 ± 0.10	183.45 ± 1.85	− 180.46 ± 1.83
PA/CNTs-*g*-PA1	59.42 ± 0.10	58.97 ± 0.10	214.23 ± 2.03	− 213.38 ± 2.17
PA/CNTs-*g*-PA2	60.00 ± 0.10	59.57 ± 0.11	224.26 ± 1.98	− 222.78 ± 2.34
PA/CNTs-*g*-PA3	59.61 ± 0.11	58.45 ± 0.10	230.18 ± 2.01	− 228.96 ± 2.34
SA/CNTs-*g*-SA1	63.82 ± 0.11	65.08 ± 0.11	235.37 ± 2.23	− 233.21 ± 2.09
SA/CNTs-*g*-SA2	64.73 ± 0.12	65.92 ± 0.12	243.25 ± 2.52	− 242.43 ± 2.47
SA/CNTs-*g*-SA3	64.90 ± 0.10	65.12 ± 0.11	256.67 ± 2.63	− 255.56 ± 2.61
CNTs/CA	28.07 ± 0.10	26.54 ± 0.13	171.43 ± 1.73	− 171.34 ± 1.73
CNTs/PA	59.70 ± 0.12	59.10 ± 0.10	210.67 ± 2.13	− 209.53 ± 2.13
CNTs/SA	66.50 ± 0.11	66.90 ± 0.10	233.86 ± 2.23	− 233.38 ± 2.43

### Cycling phase change reliability and chemical stability of the composite PCMs

**Figure 10:**
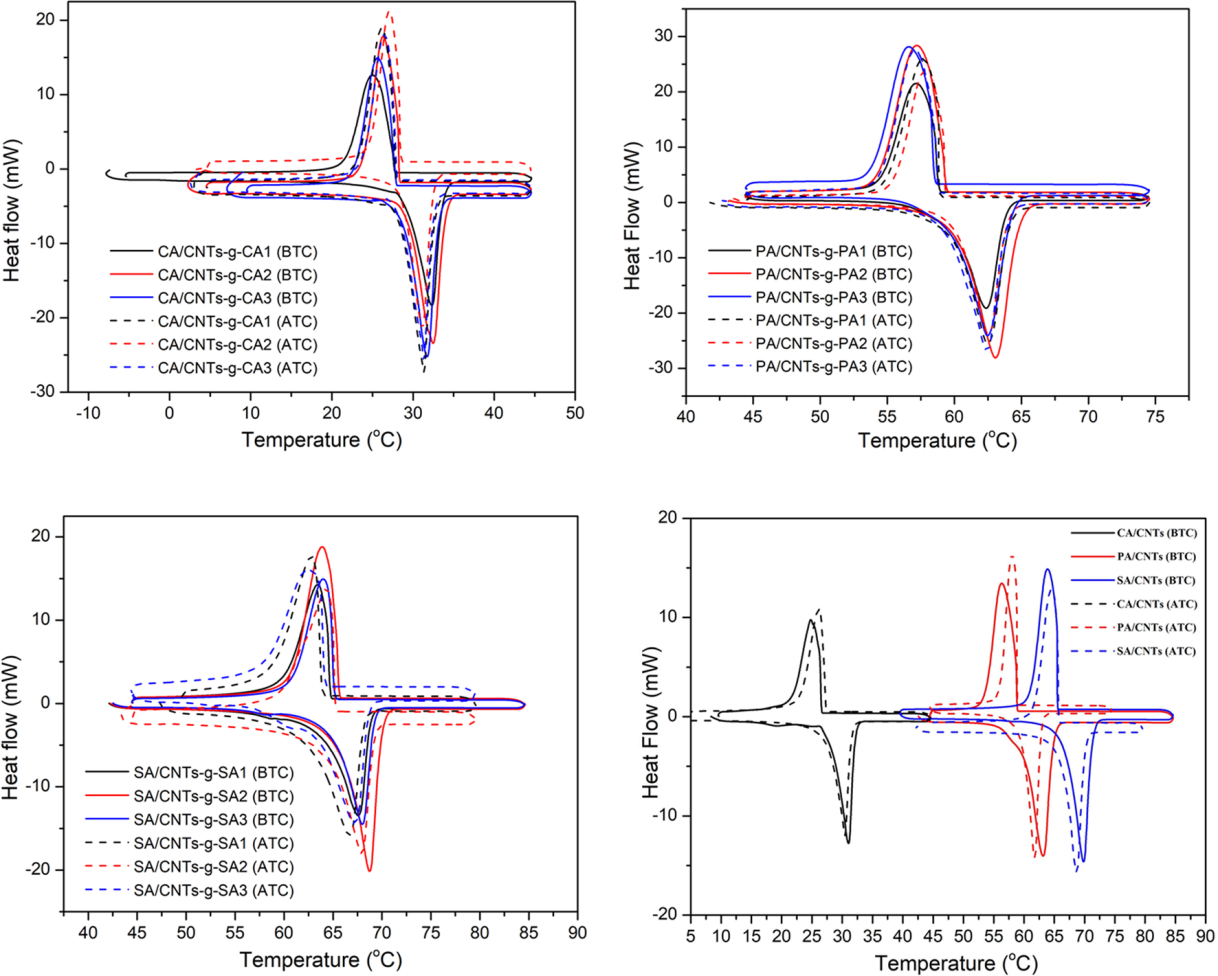
DSC thermograms of FA/CNTs-*g*-FA and FA/CNTs composites before and after 2000 cycles.

**Figure 11:**
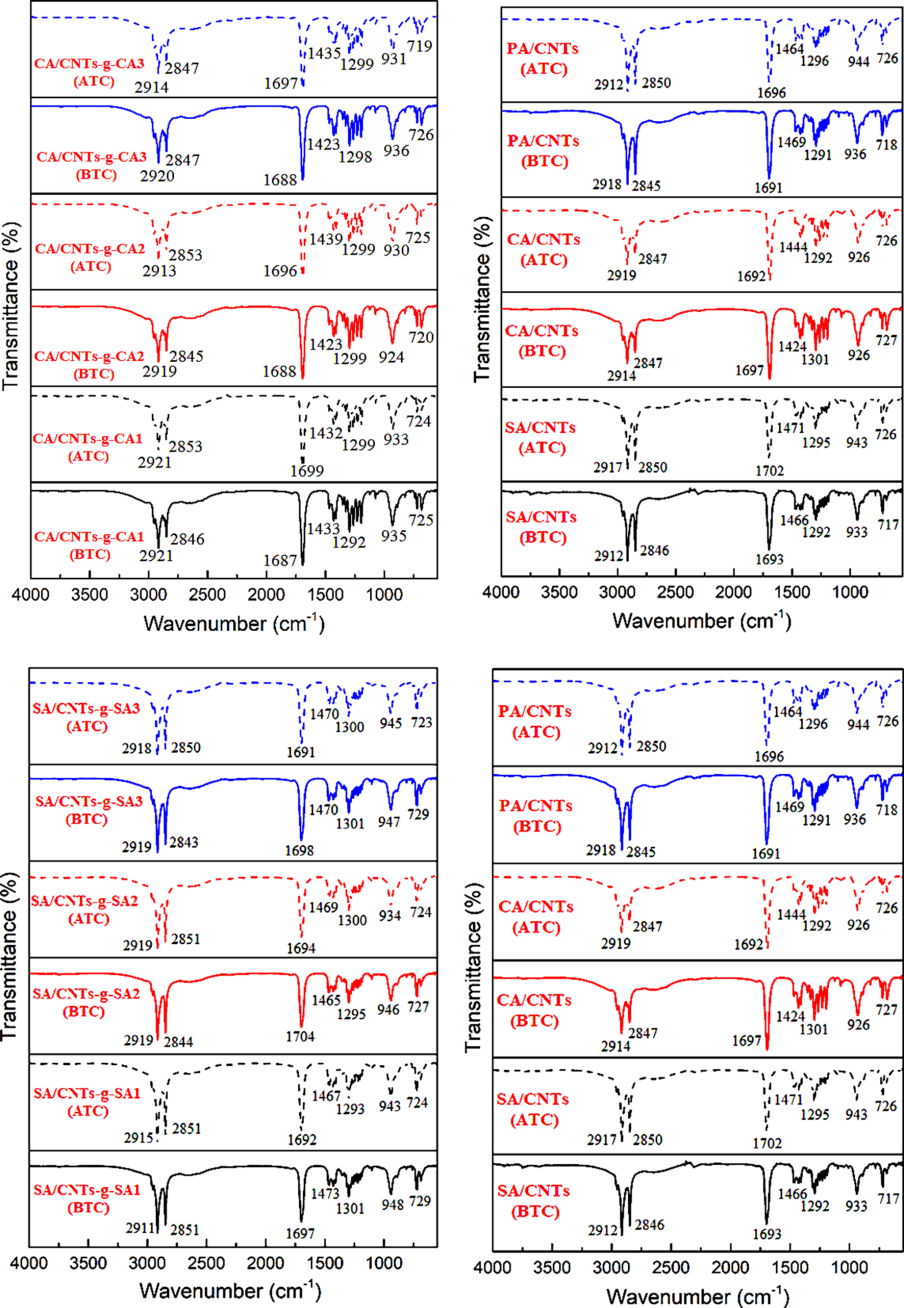
FTIR results of FA/CNTs-*g*-FA and FA/CNTs composites before (BTC) and after (ATC) 2,000 cycles.

The cycling phase change reliability is one of the major characteristic preference of a PCM. Therefore, it is desired that the PCM should have stable phase change behavior after considerable number of heating–cooling cycles^24,28^. Based on this criterion, a cycling test including consecutive cycling process repeated for 2000 times was conducted with the FA/CNTs-*g*-FA (5 wt%) and CNTs/FA (5 wt%) composites. Figure 10 shows the DSC results of the samples after the 2,000 cycles, and all LHTES data are summarized in Table 3. By considering the phase change temperature data, it was realized that there was a slight reduction in phase change temperature. The changing quantity was calculated between − 1.22 °C and 0.40 °C for melting temperature and between − 1.20 °C and 0.93 °C for solidification temperature of FA/CNTs-*g*-FA composites. As for the CNTs/FA composites it was determined in the range of (− 0.11) to (− 0.70) °C and (− 0.90) to (+ 0.93) °C for melting and solidification period . On the other hand, the reduction quantity in the LHC values of all FA/CNTs-*g*-FA and CNTs/FA were observed to be in the same level. The decrease in LHC of FA/CNTs-*g*-FA samples was changed from 10.1 to 14.2% for melting period and from 6.5 to 13.6% for freezing period. Whereas, it was varied in the range of 9.1–12.5% for melting period and 7.7–13.6% for freezing period of the CNTs/FA composite. By overall evaluation of these results, it can be concluded that the fluctuations in the TES values of all composites were in tolerable range and they had appreciable phase change stability even after 2,000 cycles. Figure 11 compares the FTIR spectra of FA/CNTs-*g*-FA (5 wt%) and CNTs/FA (5 wt%) composites before and after the 2,000 cycles. There was no significant shift of peaks were observed in the wavenumber characteristic absorption peaks of all the composites. Additionally, no extra band was observed after the cycling study. This indicates that all the prepared composite PCMs had adequate chemical stability.

**Table 3:** The LHTES properties of FA/CNTs-*g*-FA and FA/CNTs composites after 2000 thermal cycles.

Material	Melting temperature (°C)	Change (°C)	Solidification temperature (°C)	Change (°C)	Melting enthalpy (J g^−1^)	Reduction (%)	Solidification enthalpy (J g^−1^)	Reduction (%)
CA/CNTs-*g*-CA1	28.27 ± 0.13	− 0.58	27.64 ± 0.12	− 0.43	151.21 ± 1.53	13.2	− 151.12 ± 1.53	12.9
CA/CNTs-*g*-CA2	28.60 ± 0.10	− 0.83	28.80 ± 0.12	0.23	154.13 ± 1.57	13.9	− 155.34 ± 1.56	13.1
CA/CNTs-*g*-CA3	28.40 ± 0.10	− 0.44	28.16 ± 0.10	0.21	157.45 ± 1.53	14.2	− 158.56 ± 1.66	12.1
PA/CNTs-*g*-PA1	59.82 ± 0.11	0.40	58.99 ± 0.11	0.02	192.67 ± 1.94	10.1	− 199.50 ± 2.13	6.5
PA/CNTs-*g*-PA2	60.01 ± 0.11	0.01	59.43 ± 0.10	− 0.14	193.54 ± 1.96	13.7	− 192.33 ± 1.97	13.6
PA/CNTs-*g*-PA3	59.39 ± 0.11	− 0.22	58.81 ± 0.13	0.36	201.34 ± 1.99	12.5	− 199.54 ± 2.13	12.8
SA/CNTs-*g*-SA1	62.60 ± 0.10	− 1.22	63.88 ± 0.11	− 1.20	204.34 ± 2.03	13.2	− 203.32 ± 2.08	12.8
SA/CNTs-*g*-SA2	64.61 ± 0.12	− 0.12	65.21 ± 0.10	− 0.71	217.27 ± 2.18	10.7	− 215.04 ± 2.23	11.3
SA/CNTs-*g*-SA3	64.60 ± 0.11	− 0.30	64.20 ± 0.11	− 0.92	221.19 ± 2.31	13.8	− 219.67 ± 2.23	11.4
CNTs/CA	27.40 ± 0.12	− 0.67	27.47 ± 0.10	0.93	151.33 ± 1.53	11.7	− 153.01 ± 1.57	10.6
CNTs/PA	59.59 ± 0.10	− 0.11	58.88 ± 0.12	− 0.22	191.45 ± 1.87	9.1	− 193.32 ± 1.98	7.7
CNTs/SA	65.80 ± 0.11	− 0.70	66.00 ± 0.10	− 0.90	204.67 ± 2.03	12.5	− 201.54 ± 2.18	13.6

### Thermal degradation stability of the composite PCMs

**Figure 12:**
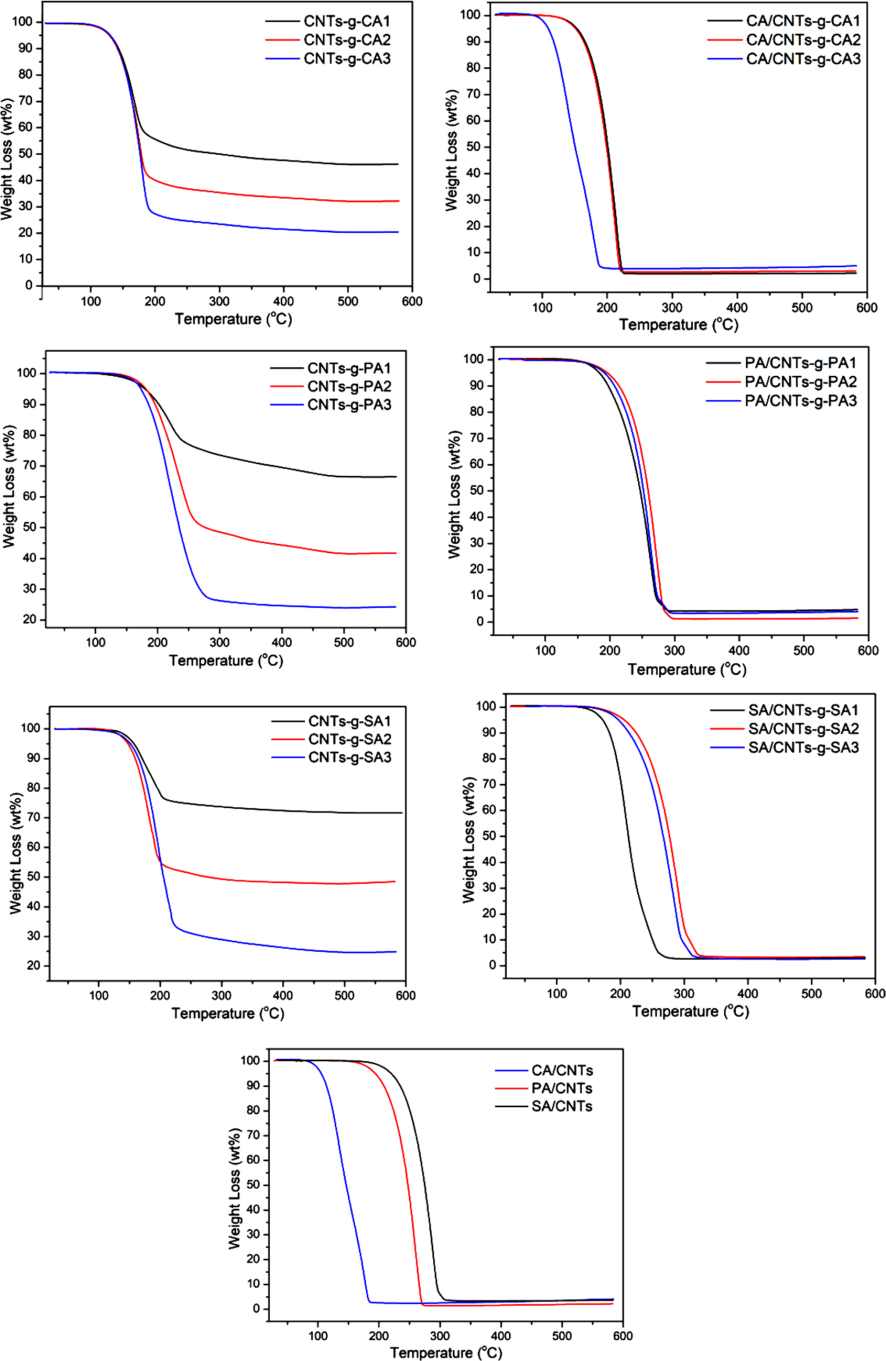
TGA thermograms of the synthesized CNTs-*g*-FA, FA/CNTs-*g*-FA and FA/CNTs.

Thermal degradation stability is one of the major thermal characteristics considered for any PCM for its suitability in a TES system. With this sense, the thermal degradation stability of the prepared composites was studied and the TGA curves were presented in Fig. 12 and Table 4. As clearly observed from the thermal curves, the prepared CNTs-*g*-FA, FA/CNTs-*g*-FA and FA/CNTs composites showed one-step thermal degradation behavior. Moreover, the grafting ratio of FA was affected thermal degradation temperature range of the final product, CNTs-*g*-FA. The degradation temperature range was determined as, 102 ± 1.06 °C–235 ± 1.12 °C; 154 ± 1.11 °C–278 ± 1.23 °C; 180 ± 1.09 °C–280 ± 1.06 °C for CA/CNTs-*g*-CA, PA/CNTs-*g*-PA and SA/CNTs-*g*-SA, while it was measured as 108 ± 1.26 °C–200 ± 1.02 °C; 162 ± 1.03 °C–293 ± 1.18 °C; 208 ± 1.22 °C–309 ± 1.21 °C for CA/CNTs, PA/CNTs and SA/CNTs, respectively. These data indicated that the thermal decomposition temperatures of the FA/CNTs samples were extended slightly. It could be due to the covalent functionalization of CNTs with FA and more effective chemical interface of FA with CNTs-*g*-FA^22^. Consequently, the prepared composite PCMs had adequately thermal degradation stability because the decomposition temperature of each composite PCM is much higher than its own phase change temperature value.

**Table 4:** Thermal degradation temperature range of CNTs-*g*-FA, FA/CNTs-*g*-FA and FA/CNTs composites.

Material	Degradation temperature (°C)
CNTs-*g*-CA1	122 ± 1.07–200 ± 1.03
CNTs-*g*-CA2	122 ± 1.13–201 ± 1.09
CNTs-*g*-CA3	122 ± 1.12–202 ± 1.10
CA/CNTs-*g*-CA1	142 ± 1.07–235 ± 1.02
CA/CNTs-*g*-CA2	142 ± 1.09–235 ± 1.10
CA/CNTs-*g*-CA3	112 ± 1.07–198 ± 1.06
CNTs-*g*-PA1	154 ± 1.07–276 ± 1.05
CNTs-*g*-PA2	154 ± 1.03–277 ± 1.09
CNTs-*g*-PA3	154 ± 1.06–278 ± 1.11
PA/CNTs-*g*-PA1	158 ± 1.08–301 ± 1.10
PA/CNTs-*g*-PA2	158 ± 1.03–305 ± 1.13
PA/CNTs-*g*-PA3	158 ± 1.05–301 ± 1.11
CNTs-*g*-SA1	148 ± 1.07–204 ± 1.12
CNTs-*g*-SA2	148 ± 1.01–206 ± 1.06
CNTs-*g*-SA3	148 ± 1.07–218 ± 1.12
SA/CNTs-*g*-SA1	160 ± 1.07–280 ± 1.06
SA/CNTs-*g*-SA2	180 ± 1.07–248 ± 1.10
SA/CNTs-*g*-SA3	180 ± 1.07–248 ± 1.03
CA/CNTs	108 ± 1.07–200 ± 1.08
PA/CNTs	162 ± 1.02–293 ± 1.09
SA/CNTs	208 ± 1.05–309 ± 1.07

### TC of the prepared PCMs

Thermal conductivity (TC) is one of the essential properties for a PCM. The higher the thermal conductivity of the PCM, the faster will be the rate of heat loading or releasing from the TES system. Based on this impression, this study is also focused on the TC enhancement of the FAs by adding CNTs-*g*-FA rather than only CNTs. Table 5 presents the measured TC values of CNTs-*g*-FA, FA/CNTs-*g*-FA and FA/CNTs as well as pure FAs. As noticed from the tabulated data (Table 5), the TC value of the pure FAs are 0.16 ± 0.01, 0.17 ± 0.01 and 0.19 ± 0.01 W m^−1^ K^−1^ respectively for the CA, PA and SA. It was boosted from 0.49 ± 0.02 to 0.82 ± 0.02 W m^−1^ K^−1^ for CNTs-*g*-CA, from 0.51 ± 0.02 to 0.89 ± 0.02 W m^−1^ K^−1^ for CNTs-*g*-PA, from 0.54 ± 0.02 to 0.93 ± 0.02 W m^−1^ K^−1^ for CNTs-*g*-SA, which depends on the increasing grafting ratio of CNTs. This increase is directly related with the higher amount of CNTs bonding of FAs. Moreover, the TC value was determined in the range of 0.24 ± 0.01–0.29 ± 0.01 W m^−1^ K^−1^ for CA/CNTs-*g*-CA (5 wt%) set of composite PCMs, 0.26 ± 0.01–0.32 ± 0.01 W m^−1^ K^−1^ for PA/CNTs-*g*-PA (5 wt%) set of composite PCMs, 0.28 ± 0.01–0.36 ± 0.01 W m^−1^ K^−1^ for SA/CNTs-*g*-SA (5 wt%) set of composite PCMs. Compared to the pure FAs, these composites showed linear increase of TC values, it was in the range of 50.0–81.3% for CA/CNTs-*g*-CA, 52.9–88.22% for PA/CNTs-*g*-PA and 47.4–89.5% for SA/CNTs-*g*-SA group of composite PCMs. On the other hand, the improvement (%) of TC values of CA/CNTs (5 wt%), PA/CNTs (5 wt%) and SA/CNTs (5 wt%) composite PCMs was calculated as 37.5, 70.6 and 68.4% compared to that of pure CA, PA and SA, respectively. In comparison to pure FAs or FA/CNTs composites, a higher degree of TC enhancement was observed from the FA/CNTs-*g*-FA composite PCMs, which was attributed to their increased interfacial compatibility/or interfacial contact area between the grafted CNTs and FA^23,29^. Relatively better miscibility and affinity between the covalent functionalized CNTs and FAs allowed improving interfacial thermal transport and thus remarkably enriching their TC values^22,26,30,31^. Accordingly, CNTs-*g*-FA acted as good filler to increase TC of the FAs by reducing interfacial thermal resistance^32,33^. Similar phenomenon was observed for the erythritol as PCM by adding acid-functionalized CNTs^21^. Additionally, the effect of the thermal cycling operation on the TC property of the produced FA/CNTs-*g*-FA and FA/CNTs composites were also investigated. As seen from the tabulated data in Table 5, the TC values was not changed in the most of composite samples, while it was slightly boosted for few samples, mainly due to slight improvement in the dispersion stability after the thermal cycling. On the other hand, the measured TC values of FA/CNTs-*g*-FA and FA/CNTs composites were compared with those of some PCMs doped with different kind of carbon based-fillers in Table 6. As seen from the tabulated data, the CPCM prepared in this work had much superior thermal properties than the previously reported similar PCMs^14,20,22,26,34–36^.

**Table 5:** The measured TC values of FA/CNTs-*g*-FA and FA/CNTs composites before thermal cycling (BTC) and after thermal cycling (ATC).

Material	TC value (W/mK) (BTC)	TC value (W/mK) (ATC)
CA	0.16 ± 0.01	Not measured
PA	0.17 ± 0.02	Not measured
SA	0.19 ± 0.01	Not measured
CNTs-*g*-CA1	0.82 ± 0.02	0.83 ± 0.02
CNTs-*g*-CA2	0.66 ± 0.02	0.66 ± 0.02
CNTs-*g*-CA3	0.49 ± 0.02	0.49 ± 0.02
CA/CNTs-*g*-CA1	0.29 ± 0.01	0.29 ± 0.01
CA/CNTs-*g*-CA2	0.27 ± 0.01	0.28 ± 0.01
CA/CNTs-*g*-CA3	0.24 ± 0.01	0.24 ± 0.01
CNTs-*g*-PA1	0.89 ± 0.02	0.89 ± 0.02
CNTs-*g*-PA2	0.68 ± 0.02	0.69 ± 0.02
CNTs-*g*-PA3	0.51 ± 0.02	0.51 ± 0.02
PA/CNTs-*g*-PA1	0.32 ± 0.01	0.32 ± 0.01
PA/CNTs-*g*-PA2	0.28 ± 0.01	0.29 ± 0.01
PA/CNTs-*g*-PA3	0.26 ± 0.01	0.27 ± 0.01
CNTs-*g*-SA1	0.93 ± 0.02	0.94 ± 0.02
CNTs-*g*-SA2	0.73 ± 0.02	0.73 ± 0.02
CNTs-*g*-SA3	0.54 ± 0.02	0.55 ± 0.02
SA/CNTs-*g*-SA1	0.36 ± 0.01	0.36 ± 0.01
SA/CNTs-*g*-SA2	0.30 ± 0.01	0.31 ± 0.01
SA/CNTs-*g*-SA3	0.28 ± 0.01	0.29 ± 0.01
CA/CNTs	0.22 ± 0.01	0.22 ± 0.01
PA/CNTs	0.29 ± 0.01	0.30 ± 0.01
SA/CNTs	0.32 ± 0.01	0.32 ± 0.01

**Table 6:** Comparison of the TC values of some organic PCMs doped with modified and non-modifed carbon based fillers.

PCM	Carbon based filler	Doping amount (wt%)	Increase in TC (%)	References
Stearic acid (SA)	CNTs	2.0	61.5	^14^
SA	CNTs	6.0	92.3	^14^
SA	CNTs	10.0	119.2	^14^
PA	CNTs	1.0	36.44	^20^
PA	Oxidized CNTs	1.0	39.25	^20^
PA	Grafted CNTs	1.0	34.11	^20^
Paraffin	Tetradecyl alcohol-g-CNT	4.0	239.13	^22^
Paraffin	Stearyl alcohol-g-CNT	4.0	243.47	^22^
Paraffin	Octanol-g-CNTs	4.0	234.78	^22^
Lauric acid(LA)	LA-g-graphene aerogel	5.0	352.10	^26^
Cetyl alcohol/HDPE	Carbon nanofiber	5.0	256.68	^34^
Stearyl alcohol/HDPE	Expanded graphite	3.0	240.69	^35^
Octadecanol	Graphene	1.5	44.35	^36^
CA	CNTs-g-CA	5.0	81.30	This work
PA	CNTs-g-PA	5.0	88.22	This work
SA	CNTs-g-SA	5.0	89.50	This work
CA	CNTs	5.0	37.50	This work
PA	CNTs	5.0	70.60	This work
SA	CNTs	5.0	68.40	This work

## Conclusions

In the present study, CNTs was functionalized and three FAs (CA, PA and SA) were grafted separately at three different ratios. FTIR, SEM and XRD techniques were used to investigate the grafting reactions, the surface modification of CNTs, compatibility and crystallites of the components. The synthesized materials were then used as additive in a mass fraction of 5 wt% to prepare respective composites to achieve good LHC and at the same time to boost the TC of the PCMs. The TES properties, thermal degradation temperatures and TC values of all prepared composite PCMs were measured. Moreover, their cycling phase change reliability and chemical structure stability were studied. The chemical structure and crystallites of the CNTs-*g*-FA, FA/CNTs-*g*-FA and FA/CNTs were confirmed by FTIR and XRD, and the surface morphologies of the different composite PCMs were evaluated by SEM. The melting temperatures of the composites containing 5 wt% non-grafted and FA grafted CNT additives were measured in the range of about 28–67 °C and 29–65 °C, respectively. While their corresponding LHC values of about 171–234 J g^−1^ and 183–257 J g^−1^, respectively. After 2000, cycles, the melting temperature of FA/CNTs-*g*-FA composites was changed between − 1.22 °C and 0.40 °C, as it was determined in the range of (− 0.11) to (− 0.70) °C and CNTs/FA composites. The cycling results also revealed that all composite PCMs had appreciable phase change stability and good chemical stability even after 2,000 cycles. The composites including grafted CNTs and non-grafted CNTs additives were thermally degraded in temperature interval of around 102–280 °C and 108–309 °C, respectively. These data proved that the composites had satisfactory thermal stability. Compared to the pure FAs, the TC values of the composite PCMs were increased in the range of about 50–90% by adding CNTs-*g*-FA in mass fraction of 5 wt%, while the TC values of the composites were boosted in the range of about 38–68% in case of adding same amount of pure CNTs. The relatively higher improvements in TCs and LHCs of the composites doped with grafted CNTs were due to the better dispersion and affinity compared to non-grafted CNTs. In addition, thermal cycling treatment had an insignificant effect on the TC values of all composite PCMs. Consequently, good dispersion stability, phase change temperatures, LHC properties, enhanced TC values, cycling thermal/chemical stability and high thermal degradation temperatures make the FAs doped with CNTs-*g*-FA additive very suitable large-scale practice TES applications.
